# Prestretched airway smooth muscle response to length oscillation

**DOI:** 10.14814/phy2.13076

**Published:** 2017-01-26

**Authors:** Ahmed M. Al‐Jumaily, Kevin Roos, Sandy Bessaguet, Miguel Jo Avila

**Affiliations:** ^1^ Institute of Biomedical TechnologiesAuckland University of TechnologyAucklandNew Zealand

**Keywords:** ASM, asthma, CPAP, prestretch, superimposed length oscillation

## Abstract

Airway smooth muscle (ASM) hyperconstriction is the cause of many respiratory diseases including asthma. In vitro testing has demonstrated that the active forces of ASM are reduced by length oscillation (LO) mimicking tidal breathing. In a previous study, we demonstrated that this force reduction can be further enhanced when superimposing oscillations (with certain frequencies and amplitudes) on this LO. In contrast, it has been reported that pressurizing the lung may help in relieving asthmatic airway constrictions. Ultimately, this pressurizing stretches the ASM and may disturb the acto‐myosin cross‐bridges in a manner similar to LO; however, it is of a static rather than dynamic nature. This research investigates the effect of combining both prestretch‐ and LO‐applications on contracted porcine ASM. Isolated porcine ASM relaxation was tested with a 0.56%, 2%, or 4% stretch of its reference length (*L*_ref_) in addition to LO. These oscillations are composed of a main wave mimicking the normal breathing (frequency of 0.33 Hz and amplitude of 4% *L*_ref_) and superimposed oscillations (frequencies of 20, 30, 40, 60 and 80 Hz and amplitude of 1% *L*_ref_). The oscillations were maintained for 10 min. The results demonstrate that a prestretch of 0.56% and 2% *L*_ref_ does enhance the contracted ASM relaxation at certain superimposed length oscillations frequencies while of 4% *L*_ref_ does not.

## Introduction

According to the World Health Organization, about 235 million people were suffering from asthma in 2013. This disease is a public health problem in all countries regardless of the level of development (Beasley 2002; Enfumosa 2003). Asthma symptoms are mainly due to bronchoconstriction of the airways. It is recognized that airway smooth muscle (ASM) contraction is a major cause for asthma obstruction and that it plays a role in airway remodeling and inflammation in asthma. Thus, ASM is targeted in the treatment of this disease.

Currently, bronchodilators and corticosteroids are used to treat asthma, but they are variable in effectiveness. Moreover, most inhalers, designed to prevent asthma attacks, are associated with several side effects (Pandya et al. 2014). In an attempt to look at an alternative safer treatment method, various researchers have studied isolated ASM in vitro to explain its physiological‐mechanical behavior and to understand the relaxation process of constricted airways occurring during an asthma attack. The form of relaxation considered in this study is due to applied mechanical length oscillation (LO) to the ASM.

Previous studies have been conducted on isolated ASM's response to LO. One of the most significant outcomes is the fact that imposing tidal breathing oscillations on the muscle induces relaxation after a chemical‐induced contraction (Du et al. 2007; Al‐Jumaily et al. 2012, 2015). Many studies have also shown that deep inspiration (DI) has a bronchodilating and a bronchoprotective effect on the ASM (Kapsali et al. 2000; Scichilone et al. 2000; Gunst et al. 2001). Bronchodilation effects are usually attributed to unconfirmed disruption of acto‐myosin cross‐bridges (Fredberg et al. 1999), while bronchoprotection (when DI is applied before the contraction) is attributed to the plasticity of the smooth muscle; however, their mechanisms remain unknown (Wang et al. 2000; Wang and Paré 2003). However, asthmatic and healthy subjects' responses to DI are different. In severe disease cases, DI can even induce bronchoconstriction instead of bronchodilation. This is attributed to a failure of DI to stretch and relax the ASM of asthmatic subjects. Burns and Gibson (1998) found that even in the absence of DI, asthmatic subjects were more responsive than healthy subjects and concluded that the hyperresponsiveness in asthma cannot be attributed entirely to an abnormal response to DI. Brown and Mitzner (2003) found that DI of short durations (5 or 10 sec) resulted in the constriction of dog ASM contracted by metacholine, while longer durations of DI (30 or 60 sec) resulted in airway dilation. Later, Slats et al. (2008) reported that positive‐pressure inflation of the lungs enhanced the bronchodilation of asthmatic patients' airways compared with active DI. These findings indicate that the way in which mechanical strains and their durations are applied on the airway have a determinant effect on airway responsiveness. On the other hand, Xue et al. (2011) found that chronic mechanical distension of the respiratory system using continuous positive airway pressure (CPAP) reduces the airway responsiveness in vivo in an allergen‐induced rabbit model of asthma. They also noticed that isolated airways from rabbits subjected to chronic CPAP were hyporesponsive in vitro and that the amount of ASM within the airway wall was not reduced by the use of CPAP. Thus, they concluded that CPAP has a direct effect on ASM contractility. The underlying mechanism remains unknown.

Al‐Jumaily et al. (2012) investigated the effect of superimposed length oscillations (SILO) in combination with tidal breathing oscillations, on isolated ASM. Their data reported that both tidal breathing oscillations alone and SILO on tidal breathing reduced the active force generated by ASM from healthy airways, and that the latter generated even better relaxation. Jo‐Avila et al. (2015) were the first to study the effect of SILO on tidal breathing oscillations on ASM from sensitized mice. They showed that SILO with specific amplitude and frequency ranges on breathing oscillations increase the relaxation of the sensitized airways compared with breathing oscillations alone.

From the above, it seems there is merit in combining the reduction in the airway responsiveness by CPAP (Xue et al. 2011) and the SILO (Al‐Jumaily et al. 2012) to see if this combined effect can produce better bronchodilation. This study is undertaken to investigate the effect of prestretching the ASM (mimicking a continuous positive pressure on the airway) before applying SILO combined with breathing oscillations. Indeed, from a mechanical perspective, a positive pressure applied on the airways induces a circumferential stretch of the inside of the airway walls. This circumferential stretch can be assumed equivalent to a longitudinal stretch of the isolated ASM bundles. Physiologically, this type of stretch may generate some passive length relaxation which could be attributed to length adaptation in ASM while LO may contribute to the disturbance of cross‐bridge cycling. Each one of these individually contribute to ASM relaxation (Ijpma et al. 2011). The question raised here is would combining them have an additive effect to relaxation.

## Materials and Methods

### Tissue preparation and experimental apparatus

Porcine tracheas were acquired from Auckland Meat Processors at the time of animal processing. Trachea were cleaned and stored in an airtight container with physiological Krebs solution at 4°C over 2–3 days. The physiological solution (NaCl: 110.54 mmol/L, KCl: 3.39 mmol/L, KH_2_PO_4_: 1.20 mmol/L, MgSO_4_ pure: 0.82 mmol/L, d(+) glucose monohydrate: 5.15 mmol/L, NaHCO_3_: 25.68 mmol/L, CaCl_2_ 2H_2_O: 2.40 mmol/L) was changed every day.

ASM strips were dissected from the trachea. Epithelium and connective tissues were carefully removed in the circumferential direction and small strips of tissue (1 × 0.5 × 10 mm) were dissected from the muscle sheet in the same direction. While one end of the strip was knotted with a single loop of silk thread and later immobilized on a motionless glass hook, the other end was knotted with silk thread for attachment to a servo‐controlled lever arm (Model 300B; Cambridge Technology, Aurora Scientific Aurora, ON, Canada) in order to establish the length of the tissue strip. The whole muscle bundle was suspended vertically in a 5‐mL bath filled with the physiological Krebs solution, bubbled with carbogen (95% O_2_ and 5% CO_2_) to maintain the pH at 7.4, and kept at 37°C using a surrounding water jacket. All output data (Force‐ and Length‐ controlled) were acquired and sent to a National Instruments LabVIEW program, which was developed earlier for this purpose.

### Experimental protocol

ASM bundles were equilibrated in physiological solution and then stretched progressively to an optimal length called the reference length (*L*_ref_). Muscle strips underwent several cycles of acetylcholine‐induced contraction ([ACh] = 10^−4^ mol/L), washing to remove ACh, and incremental stretches (not more than 5% of the current length) to finally reach the optimal length. Optimal length was reached when the generated force (the difference between the maximal force plateau and the force measured before the addition of ACh) was similar between successive contractions. Experiments were then conducted at this length. Prior to initiating the protocols, the ASM was allowed to rest for about 20 min to re‐equilibrate and reach a baseline tension. Two protocols were paired in order to assess the effect of prestretching prior to application of mechanical oscillations.

**Figure 1:**
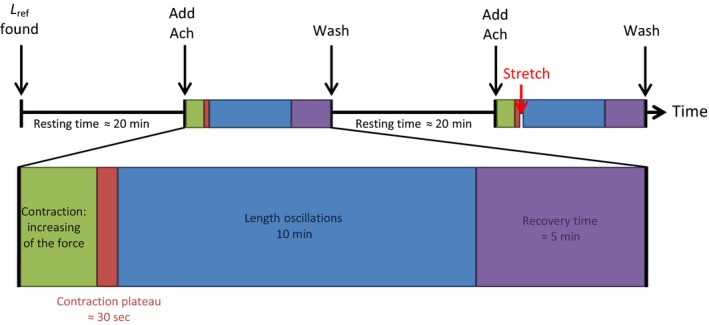
Protocol diagram.

In the first protocol, the muscle bundle was maximally contracted using ACh, and when a stable force plateau was observed (for at least 30 sec), mechanical oscillations were then applied for 10 min. A recovery time of 5 min was set to let the muscle recover from the oscillations. At 5 min of this recovery time, the final force was measured and the tissue was washed by replacing fresh physiological buffer to the system. After a 20‐min rest, the second protocol was then applied to the tissue. The ASM was contracted with ACh as in the first protocol. Once the force plateau was observed, the muscle bundle was initially stretched to either 0.56%, 2%, or 4% of the *L*_ref_. Oscillations similar to those in the first protocol were then applied to the strip after the prestretch. Again, a recovery time of 5 min was given to the muscle before measuring the final force while the muscle was still under the initial stretching condition; see Figure 1.

The applied oscillations used in the protocols are combinations of breathing cycles plus SILO. Oscillations used to mimic porcine normal breathing in vitro are at a frequency of 0.33 Hz with an amplitude of 4% of the *L*_ref_. The SILO were at several frequencies (20, 30, 40, 60, and 80 Hz) with an amplitude of 1% of the *L*_ref_. In all of the tests, the muscle bundle was considered damaged and discarded when the subsequent force was lower than 75% of the previous active force.

### Result analysis

The forces measured before and after applied mechanical treatments were normalized with respect to the generated force induced by ACh (i.e., the force measured from the force plateau obtained before applying stretch and/or LOs). The statistical tests were conducted using two‐way analysis of variance (ANOVA) with the level of acceptance set at the probability *P* < 0.05.

## Results

**Figure 2:**
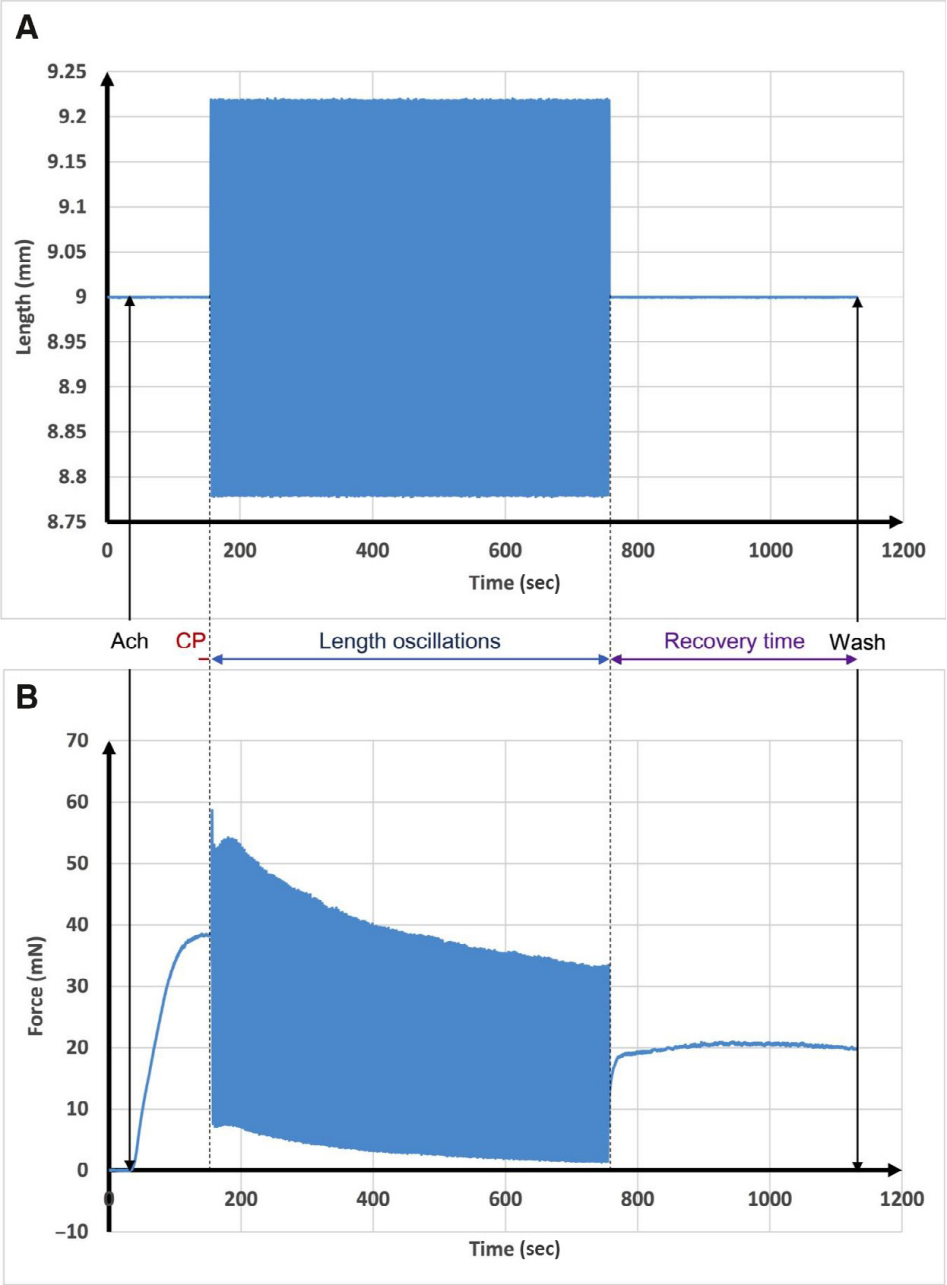
Typical output data obtained from a standard protocol without stretch and with length oscillations. (A) Length‐time curve applied to the ASM. (B) Force‐time curve response to those length oscillations. ASM, airway smooth muscle; CP, contraction plateau.

**Figure 3:**
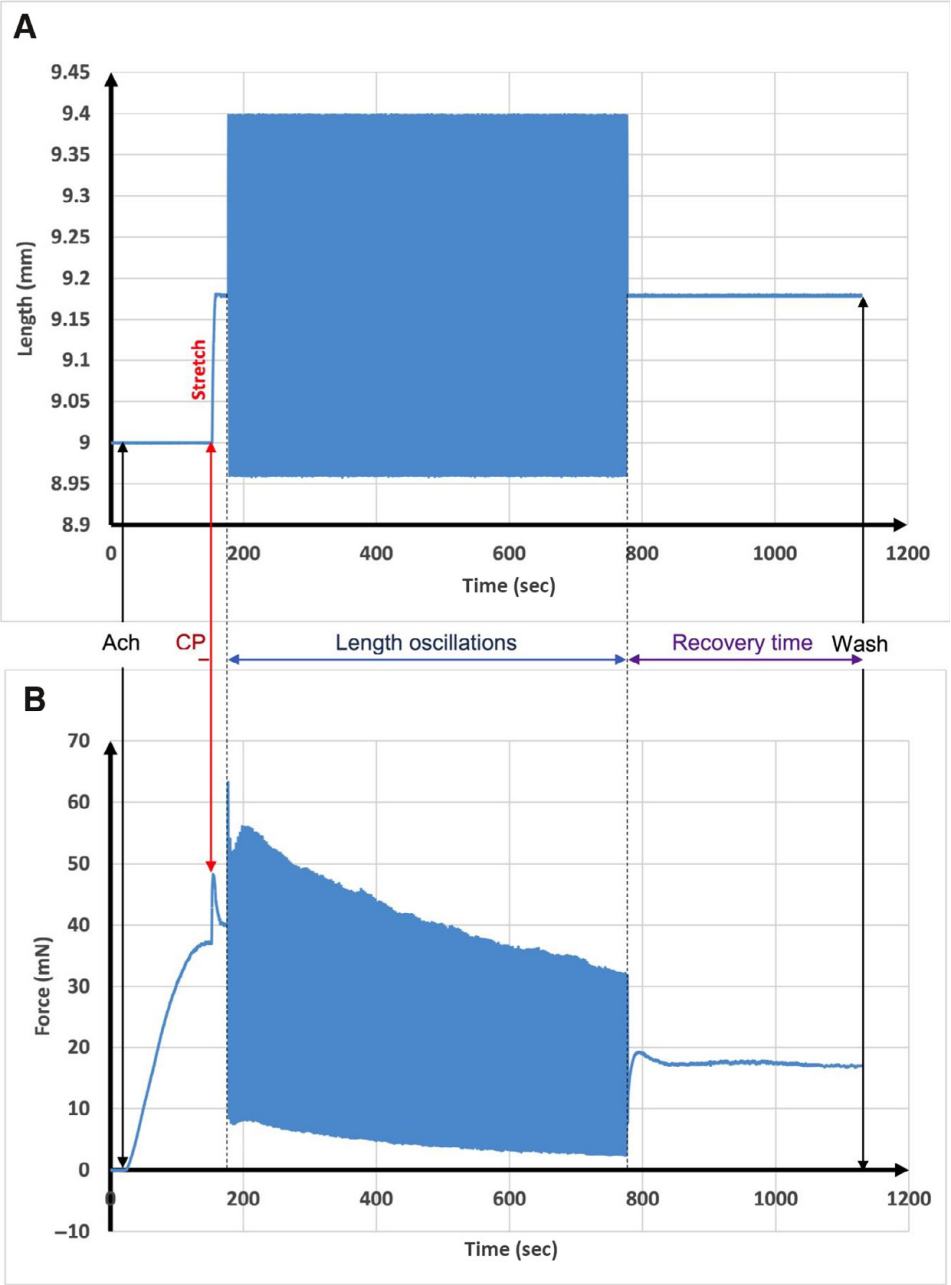
Typical output data obtained from a protocol with stretch and length oscillations. (A) Length‐time curve applied to the ASM. (B) Force‐time curve response to those length oscillations. ASM, airway smooth muscle; CP, contraction plateau.

Figure 2 shows a typical trace of the length‐time applied without a prestretch during the first protocol and the corresponding force‐time response. In this case, the tissue is undergoing simultaneous tidal oscillation of 4% *L*_ref_ amplitude and 0.33 Hz frequency combined with SILO of 1% *L*_ref_ amplitude and 30 Hz frequency. Figure 3 shows a similar trace, but with a prestretch of 2% *L*_ref_ according to the second protocol. It is evident from these curves that the SILO produce significant reduction in the recovery force after both prestretch and no prestretch conditions. However, the prestretch condition results in a lower recovery force. Obviously, lower recovery force indicates more ASM relaxation.

**Figure 4:**
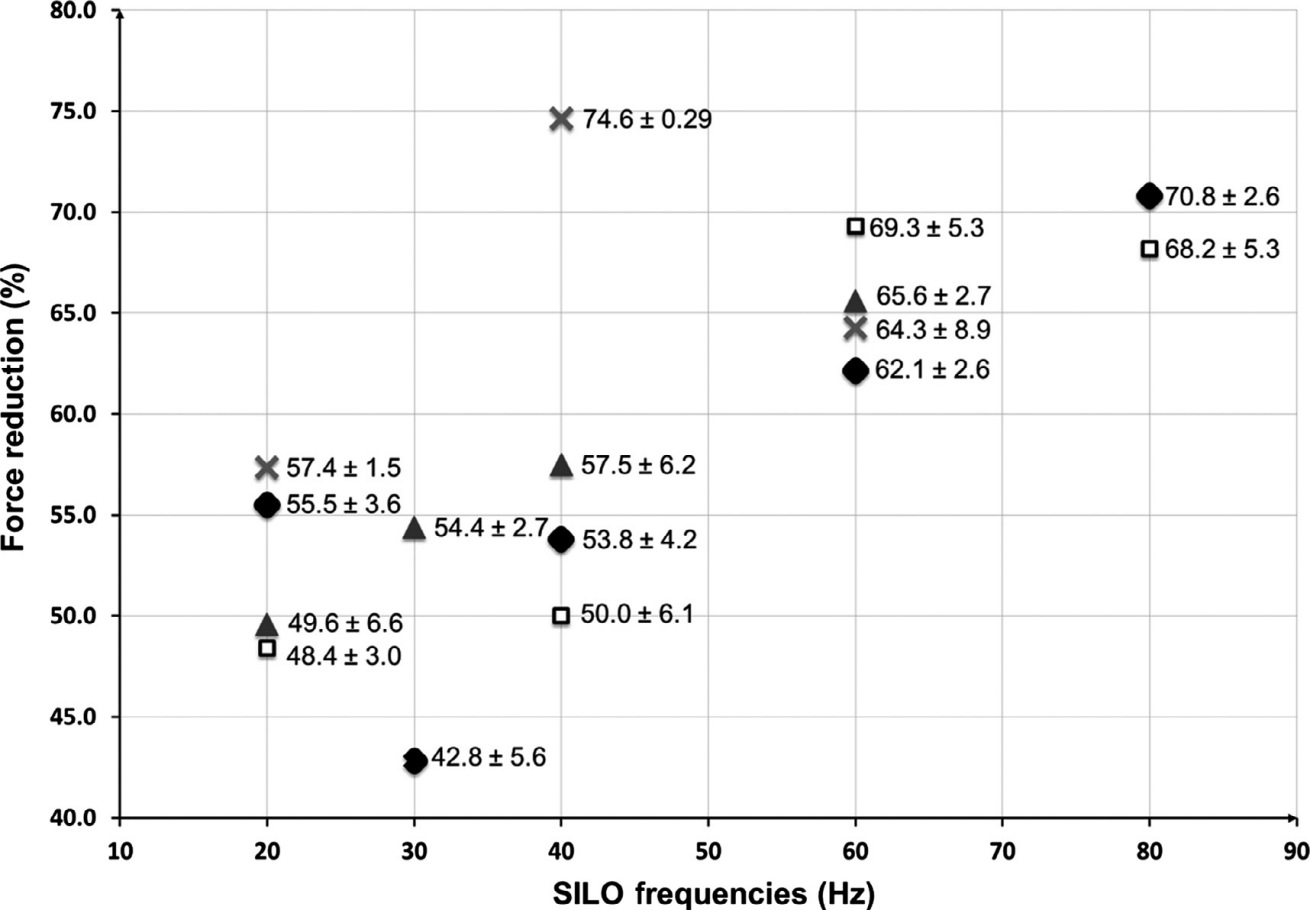
Percentage of force reduction resulting from the effect of superimposed length oscillations (20–80 Hz, and 1% amplitude of the *L*_ref_) on normal breathing conditions (0.33 Hz, 4% amplitude) applied to porcine tracheal smooth muscle tissue. Active force measurements were taken 5 min after the cessation of applied oscillations. *N*‐values for No‐Stretch (♦) controls: 20 Hz, *N* = 7; 30 Hz, *N* = 4; 40 Hz, *N* = 5; 60 Hz, *N* = 7; 80 Hz, *N* = 4. *N*‐values for 0.56% Stretch (**X**): 20 Hz, *N* = 3; 40 Hz, *N* = 3; 60 Hz, *N* = 3. *N*‐values for 2% Stretch (▲): 20 Hz, *N* = 3; 30 Hz, *N* = 3; 40 Hz, *N* = 4; 60 Hz, *N* = 4. *N*‐values for 4% Stretch (**□**): 20 Hz, *N* = 4; 40 Hz, *N* = 3; 60 Hz, *N* = 4; 80 Hz, *N* = 6. For clarity, the mean and error values are indicated at each point without error bars.

To determine the frequency dependence of SILO on the constricted ASM without and with the prestretch, an amplitude of 1% *L*_ref_ in the range of 20–80 Hz was superimposed on tidal LOs for various prestretch values. Figure 4 shows the mean value ± percentage error without and with various prestretches. For clarity, percentage errors are used rather than error bars. The figure summarizes the effect of SILO on the active force after 5 min oscillation cessation of various frequencies with 4%, 2%, and 0.56% *L*_ref_ prestretch. Force values are expressed as a percentage of the force plateau before applying the LOs. The number of samples, *N*, varies between the various tests due to the fact that in several cases, the muscle bundle was considered damaged and discarded when the subsequent force was lower than 75% of the previous active force; however, the minimum value of *N* was taken as 3.

Figure 4 indicates that a prestretch of 4% *L*_ref_ with 20, 40, 60, or 80 Hz SILO frequency has no trend in the reduction of the active force after oscillation cessation. On the contrary, the 2% *L*_ref_ prestretches have induced significant relaxation when applied at 30, 40, and 60 Hz SILO frequency; however, there is no clear relaxation at SILO frequency of 20 Hz. This figure suggests the existence of a minimum and a maximum frequency threshold settled in between 20 and 60 Hz (without including the lower limit) when the experiment was run at 2% *L*_ref_ prestretch. Additionally, the 0.56% *L*_ref_ prestretch shows significant relaxations when used with all tested SILO frequencies.

To compare the effect of these three different stretches, the normalized final force obtained from the prestretched muscle was divided by the normalized final force obtained from the same muscle but not prestretched. This figure quantifies the percentage improvement in relaxation. Evidently small prestretches such as the 0.56% *L*_ref_ show positive improvements up to 20% at 60 Hz SILO frequency, while no definite improvement is observed at large prestretches such as the 4% *L*_ref_. Analysis using two‐way ANOVA, where *α* = 0.05, established a relationship between SILO frequencies and the applied stretches with the observed reductions in ASM force. There is a significant difference in the observed percentage of force reduction for tissues that were stretched 0.56% *L*_ref_ and 4% *L*_ref_ and then oscillated with SILO frequencies of 20, 40, and 60 Hz, and 20, 40, 60, and 80 Hz, respectively. There is no significant difference, however, in the observed reductions when comparing 2% against the control set and 4%, or the 0.56% set with 2% across SILO of 20, 40, and 60 Hz. In this respect, the 2% data set is comparable to control data, while 0.56% and 4% stretch data indicate utility in reducing the force of mechanically oscillated ASM. The two‐way ANOVA statistical testing allows us to conclude that the means of the results from the different stretches in addition to same LOs are different (*P* = 0.00025) and that the means from the different SILO's frequency (different LOs) in addition to same stretches are also different (*P* = 0.0076). Overall, the comparisons indicate that there is significant interaction between the stretches and the LOs (*P* = 0.029) (Table 1).

**Table 1:** Two‐way ANOVA statistical tests

Comparison of means two‐way ANOVA	Interaction *P*‐value	Treatments *P*‐value (with or without stretch)	SILO frequencies *P*‐value
No stretch versus 4% stretch	0.025	0.899	1.75 × 10^−5^
No stretch versus 2% stretch	0.069	0.254	0.001
No stretch versus 0.56% stretch	0.045	0.086	0.013
4% versus 2% versus 0.56% stretch	0.029	0.00025	0.0076

ANOVA, analysis of variance; SILO, superimposed length oscillations.

## Discussion

Many in vitro studies have emphasized the relaxation effect of LOs similar to those which occur during normal breathing and DIs (Kapsali et al. 2000; Scichilone et al. 2000; Wang et al. 2000; Gunst et al. 2001; Wang and Paré 2003; Du et al. 2007; Al‐Jumaily et al. 2012, 2015; Jo‐Avila et al. 2015) on contracted ASM. Additionally, SILO have been previously studied and led to the conclusion that further ASM relaxation can be achieved at certain frequencies (Al‐Jumaily et al. 2012). The effect of a stretch on a contracted ASM can be deduced from the stretches applied to determine the reference length. In principle, the *L*_ref_ is determined after several stretches until a maximum stable force is reached. Additional small stretches would produce some reduction of the ASM reactive force and relaxation. This is supported by previous evidence that positive pressure of the lung enhanced bronchodilation of asthmatic patients (Slats et al. 2008) and that using CPAP reduced airway responsiveness in an allergen‐induced rabbit model for asthma. This research extends out from these findings to see whether the two actions of a prestretch and SILO have an additive nature; will they improve each other's effect or not? Thus, this study focuses on the reactivity of precontracted porcine ASM. Introduction of a permanent mechanical stretch, set between the ACh‐induced contraction plateau and the applied LOs (which mimic tidal and superimposed breathing conditions), provides the basis for the investigation. Given that SILO already proposes a method for achieving ASM relaxation, it is anticipated that the proposed prestretching of the ASM may offer an additional mechanistic condition for enhancing acto‐myosin detachment without the associated damage observed when large amplitude oscillations are applied to tissues.

This study confirms that applying SILO on breathing oscillations results in reduction of the muscle‐generated forces in a frequency‐dependent manner (Al‐Jumaily et al. 2012). However, previous findings (Wang et al. 2000; Du et al. 2007) did not confirm this dependence. This may be attributed to the fact that we used superimposed rather than pure oscillations and chemical rather than electrical activation of the muscle tissue, which in turn results in a different definition of reference length for the tissue samples.

**Figure 5:**
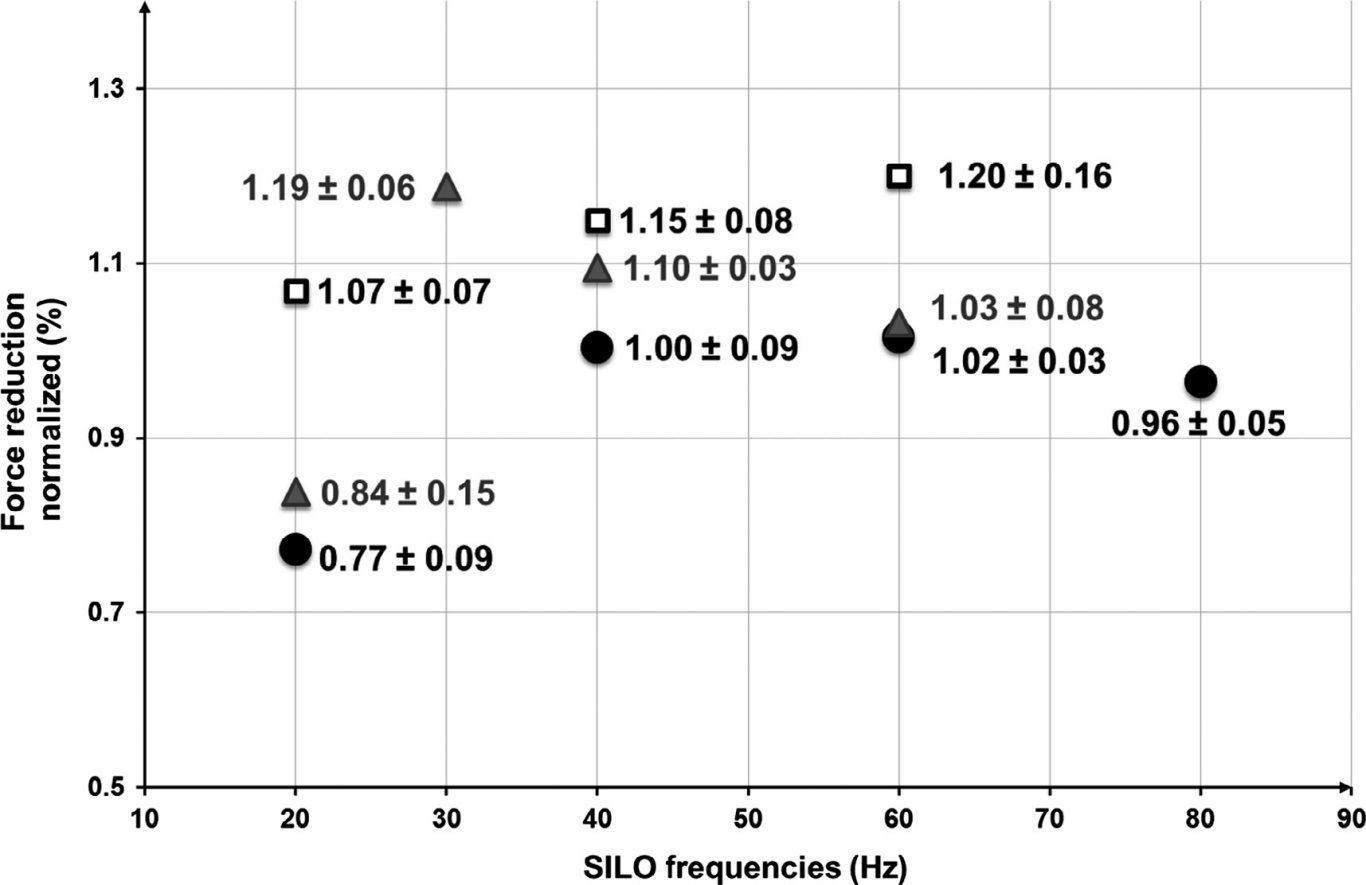
Percentage of force reduction resulting from the effect of superimposed length oscillations (20–80 Hz, and 1% amplitude of the *L*_ref_) on normal breathing conditions (0.33 Hz, 4% amplitude) applied to porcine tracheal smooth muscle tissue. Active force measurements were taken 5 min after the cessation of applied oscillations. *N*‐values for No‐Stretch controls: 20 Hz, *N* = 7; 30 Hz, *N* = 4; 40 Hz, *N* = 5; 60 Hz, *N* = 7; 80 Hz, *N* = 4. *N*‐values for 0.56% Stretch (**□**): 20 Hz, *N* = 3; 40 Hz, *N* = 3; 60 Hz, *N* = 3. *N*‐values for 2% Stretch (▲): 20 Hz, *N* = 3; 30 Hz, *N* = 3; 40 Hz, *N* = 4; 60 Hz, *N* = 4. *N*‐values for 4% Stretch (●): 20 Hz, *N* = 4; 40 Hz, *N* = 3; 60 Hz, *N* = 4; 80 Hz, *N* = 6. For clarity, the mean and error values are indicated at each point without error bars.

It has been reported that a 4% stretch can be applied without damaging the tissue (Fredberg et al. 1999). Thus, we considered this as the terms of reference in this investigation and we looked at the effect of using smaller prestretches. Figure 4 shows that for 4% prestretch, no specific trend is followed. This may be attributed to the fact that a 4% stretch added to the 4% amplitude of oscillation will lead to an 8% stretch above the *L*_ref_, which may damage the tissue and drive it beyond its normal stretching limits. For the effect of SILO on breathing with a stretch of 2% *L*_ref_, it is evident that the prestretch improved relaxation, between the boundaries of 20 and 60 Hz frequencies. A possible explanation may be attributed to the fact that at low SILO frequencies, the whole contractile apparatus has enough time to adapt to the new lengths, allowing the muscle to keep most of the acto‐myosin cross‐bridges attached (observed as less relaxation), despite the length perturbations. The use of high SILO frequencies may not give sufficient time to the contractile apparatus to receive the length perturbations; therefore, the length adaptation process does not occur and the active force does not decrease significantly. On the contrary, a very small prestretch of 0.56%, as indicated in Figure 5, shows that positive relaxation is being added to the tissue for the three frequencies used.

Several studies have found that the effect of LOs did not last for over 30 min (Wang et al. 2000; Al‐Jumaily et al. 2012) due to the fact that the recorded force after oscillation cessation tends to recover to the stable recorded force before oscillations began. However, it has been reported that using a CPAP device as treatment support for chronic asthma in conjunction with longer duration DI, has a relaxation effect which can persist for at least 24 h after removal of the device (Brown and Mitzner 2003; Xue et al. 2011). Thus, in the future, it would be interesting to study the recovery force over periods of time longer than 5 min after the cessation of applied mechanical oscillations.

One of the most important limitations of this study is the use of isolated healthy porcine ASM. The results do not predict the reactivity of porcine ASM set within the intact trachea in vivo, nor do they address the reactivity of human ASM from a non‐asthmatic or asthmatic patient. The second important limitation of this study is that the machinery's servo‐controlled lever arm sets and controls the muscle length, but does not allow us to predict the in vivo length behavior of ASM in physiological conditions, rather we are able to monitor the in vitro force response behavior to mechanically applied length changes.

In conclusion, we present the findings that SILO applied to breathing frequency oscillations has a relaxation effect on ACh‐contracted porcine ASM. Some permanent prestretches of porcine ASM tissue strips, in conjunction with a specific range of SILO frequencies, are associated with greater reduction in the active force generated by the ASM.

## Conflict of Interest

None declared.
